# Rethinking EEG biomarkers of brain disorders: a transdiagnostic dimensional view

**DOI:** 10.1038/s41398-026-04187-z

**Published:** 2026-06-20

**Authors:** Paul Theo Zebhauser, Henrik Heitmann, Peter Henningsen, Markus Ploner

**Affiliations:** 1 Center for Interdisciplinary Pain Medicine, TUM School of Medicine and Health, Technical University of Munich, Munich, Germany; 2 Department of Neurology, TUM School of Medicine and Health, Technical University of Munich, Munich, Germany; 3 TUM-Neuroimaging Center, TUM School of Medicine and Health, Technical University of Munich, Munich, Germany; 4 Department of Psychosomatic Medicine and Psychotherapy, TUM School of Medicine and Health, Technical University of Munich, Munich, Germany

**Keywords:** Biomarkers, Neuroscience, Psychiatric disorders

## Abstract

Developing clinically useful brain-based biomarkers remains a central challenge in translational psychiatry and neurology. Traditional approaches focusing on disorder-specific signals have shown limited clinical utility. EEG, a scalable and non-invasive measure of brain function, illustrates the value of an alternative perspective: transdiagnostic and dimensional biomarker development. Here, we use low-frequency activity (LFA) as an illustrative example to demonstrate this framework. We synthesize evidence from 176 EEG studies across chronic pain, migraine, fatigue, and depression and identify increased low-frequency activity (LFA) as the most consistent alteration across studies. Crucially, this absence of disorder specificity does not diminish its clinical value. Instead, it points to shared neural dysfunction, consistent with frameworks of thalamo-cortical dysrhythmia and excitation-inhibition imbalance. These processes may underlie shared symptom dimensions, such as negative affect, cognitive dysfunction, and somatic manifestations. Accordingly, such transdiagnostic, dimensional markers could support prevention, monitoring, stratification, and neuromodulation across disorders, exemplifying precision neuroscience via mechanistically grounded, clinically actionable biomarkers.

## EEG biomarkers of brain-related disorders

Developing clinically useful biomarkers has become a central goal in clinical and translational neuroscience, with potential implications for the diagnosis, monitoring, and treatment of psychiatric and neurological disorders. Current frameworks for biomarker development, such as the FDA-NIH BEST framework [1], distinguish between diagnostic, prognostic, monitoring, and predictive biomarkers, each intended to support clinical decision-making during different stages of disease or even in preclinical states. Such biomarkers can be derived from diverse modalities, among which measures of brain function hold particular promise.

EEG offers unique advantages in this context. As a non-invasive measure of neural activity, it has advanced our understanding of brain function and dysfunction for more than a century [2]. Early discoveries, from the electrophysiological signatures of sleep to the recognition of epilepsy as a disorder of aberrant brain activity, established its clinical relevance. Today, advances in hardware, computational power, and machine learning enable a new wave of EEG-based biomarker development for neurological and psychiatric conditions [3]. Because EEG is widely available, safe, low-cost, and potentially mobile, it has exceptional potential for translation into large-scale clinical practice. Moreover, alterations in brain function detected by EEG are potentially targetable with neuromodulation techniques, underscoring its promise as a bridge between mechanism and intervention.

EEG is often recorded during the resting state and analyzed using spectral decomposition, yielding a power spectrum across canonical frequency bands (delta, theta, alpha, beta, gamma; 1–100 Hz). Traditionally, biomarker discovery has focused on power within these frequency bands as indices of neural activity. Another prominent feature of this spectrum is the alpha peak around 10 Hz, whose frequency (peak alpha frequency, PAF) can itself serve as a marker of brain function. More recent connectivity measures capture the coordination of distributed neural populations using phase-, amplitude-, or information-theoretic metrics [4], reflecting the network-level dynamics that underlie cognition and behavior. Another emerging method isolates the aperiodic (1/f-like) component of the spectrum, indexing broadband slope and offset, which are thought to reflect the balance between excitation and inhibition in large neural populations [5].

These approaches have already yielded promising biomarker candidates. For example, PAF has been shown to predict differential responses to pharmacological or brain stimulation treatments in major depressive disorder [6]. Likewise, EEG connectivity patterns can identify distinct subtypes of depression and post-traumatic stress disorder, each exhibiting different responses to psychotherapy, pharmacotherapy, and brain stimulation [7]. Such findings highlight the translational promise of EEG-based biomarkers and their potential to guide personalized treatment strategies (Box 1).

Yet challenges remain: the replicability of many candidate markers remains to be demonstrated [8]. Community-wide efforts in data sharing, standardized preprocessing pipelines, and open science practices are beginning to address these limitations [9]. Another common concern is biomarker specificity. Most approaches remain anchored in diagnostic categories such as the ICD-10 and pursue disorder-specific signals [10]. This focus is traditionally motivated by the goal of identifying diagnostic indicators that can reliably distinguish between healthy and diseased states or between different disorders. This goal is highly valuable in certain clinical contexts. However, an overly narrow focus on disorder-specificity may be misplaced: biomarkers do not need to be disease-specific to be clinically useful. This principle is well illustrated by established clinical measures such as C-reactive protein, which is sensitive to inflammation across diverse diseases yet remains highly informative when interpreted within the clinical context.

This perspective opens the door for a different strategy: transdiagnostic and dimensional biomarkers that capture neural processes shared across disorders and linked to symptom dimensions rather than categorical diagnoses. Such an approach would be in line with the dimensional Research Domain Criteria framework, which emphasizes core neural circuits and functional domains across neuropsychiatric conditions [11]. Here, *dimensional* refers to biomarkers that vary quantitatively along continua of symptom severity or functional capacity, rather than mapping onto discrete diagnostic categories. This approach does not replace disorder-specific perspectives but complements them, offering a broad and potentially powerful way to connect brain function with clinical manifestations. For example, cognitive dysfunction is a core transdiagnostic feature observed across many brain-related disorders. Yet, its expression may differ between conditions, with executive deficits being a hallmark of depression [12], whereas they are less evident in chronic fatigue [13]. This dimensional perspective provides a framework for linking common neural alterations to broad symptom dimensions observed across different disorders. In the following sections, we illustrate this principle using EEG findings from the translational neuroscience of pain, showing how a transdiagnostic, dimensional perspective can enhance both mechanistic understanding and clinical application.

Box 1 What Are EEG Biomarkers?

- *In biomarker research, resting-state EEG is recorded to capture spontaneous brain activity. Standard analyses decompose the signal according to canonical frequency bands (delta, theta, alpha, beta, gamma), extracting band-specific power and peak alpha frequency (PAF). More recent approaches assess connectivity between brain regions and quantify the aperiodic (1/f-like) component, which is thought to reflect the excitation–inhibition balance of neural populations*.
- *Applied to large datasets, these methods can support the investigation of different biomarker categories. According to the FDA-NIH BEST (Biomarkers, EndpointS, and other Tools) framework, biomarkers can serve multiple complementary functions* [1]*: diagnostic biomarkers may support accurate diagnosis and refine disease phenotyping; monitoring biomarkers enable tracking disease trajectories and treatment effects; predictive biomarkers provide insights into likely responses to interventions, prognostic biomarkers may forecast disease trajectories or the risk of future episodes; and susceptibility/risk biomarkers extend their utility to presymptomatic phases and preventive functions by identifying individuals at elevated risk before clinical manifestation*.
- *Transdiagnostic dimensional biomarkers are biological signals linked to symptom dimensions - such as negative affect or cognitive dysfunction - rather than categorical diagnoses. Observed across multiple conditions, they index shared pathophysiological processes, providing a perspective that complements disorder-specific approaches. Biomarkers of brain function are particularly promising for transdiagnostic dimensional research because they can capture shared large-scale neural activity underlying broad symptom dimensions. Moreover, EEG is a low-cost and scalable method, enabling studies of large, heterogeneous samples that are essential for transdiagnostic approaches.*

## Common EEG findings in pain, headache, fatigue, and depression

The brain plays a central role in the susceptibility, development, and maintenance of chronic pain [14], making brain-based biomarkers an important target for pain neuroscience. However, the development of robust brain-based biomarkers for pain remains limited, and their clinical utility has yet to be demonstrated [15]. Against this background, we conducted a series of systematic reviews and meta-analyses of resting-state EEG (rsEEG) in chronic pain and related conditions [16–19]. We analyzed 176 studies comprising 14705 participants to evaluate EEG alterations associated with chronic pain, fatigue, migraine, and depression. This provides an unprecedented quantitative overview of EEG alterations across these diverse but related conditions.

**Fig. 1:**
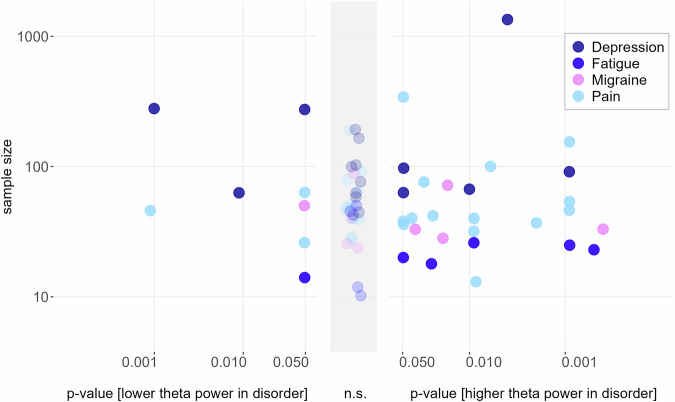
Shared Increase of Low-Frequency Activity Across Disorders. A composite albatross plot illustrates elevated theta power (4–8 Hz) across chronic pain, fatigue, migraine, and depression compared to healthy participants in 81 resting state EEG studies (*n* = 6530), suggesting a transdiagnostic neural mechanism. By plotting *p*-values against sample sizes for different directions of effects, albatross plots allow for graphically estimating effect sizes for studies with similar research questions (*e.g., is there a difference in theta activity between people with chronic pain and healthy controls?*). For migraine and depression, formal meta-analysis was feasible and yielded small to moderate effects (hedges g 0.38 and 0.22, respectively). Single data points represent individual primary studies; the x- and y-axes are log-scaled. n.s. not significant (Based on data from [16–19]).

The most consistent finding was increased low-frequency activity (LFA), particularly in the theta (4–8 Hz) range (see Fig. 1). Increased beta power (13–30 Hz) was less robust but was observed in both chronic pain and depression. Decreased alpha power was inconsistently observed across conditions. PAF was reduced in some studies of chronic pain, but yielded inconclusive results in migraine, depression, and fatigue. Connectivity analyses did not reveal consistent alterations, likely due to methodological heterogeneity across studies [20]. As risk-of-bias analyses showed limited control for potentially confounding factors (such as medication, recording conditions, or analysis choices), we cannot rule out that these factors contributed to the observed EEG changes. However, the consistent increase of LFA across conditions suggests that these factors are unlikely to be the main driver of the observed pattern.

Notably, increased LFA is not unique to these disorders: similar alterations have been reported in neurodegenerative [21, 22], neurodevelopmental, and psychiatric conditions [23]. While this lack of disorder specificity across the studied and other brain-related disorders might seem limiting, a transdiagnostic, dimensional perspective reframes it as an opportunity. Rather than indicating categorical diagnoses, LFA may reflect a fundamental neural dysfunction that cuts across diagnostic boundaries and manifests as shared symptom dimensions. Clinical decision-making often relies on such nonspecific measures: body temperature or C-reactive protein levels gain diagnostic and prognostic value when interpreted in context. In neurology, neurofilament light chain has clinical utility across disorders despite nonspecifically reflecting axonal damage [24]. Likewise, EEG measures such as LFA may contribute to mechanistic models of brain dysfunction, and, when integrated with other clinical information, support prevention, monitoring, stratification, and intervention across disorders.

## Interpreting transdiagnostic EEG findings

For a candidate biomarker, clinical utility is the primary requirement. Mechanistic interpretability is not essential, but it can increase acceptance, link findings to established knowledge, and guide targeted interventions. The example of increased LFA illustrates how a transdiagnostic biomarker can be interpreted within a mechanistic framework. Converging lines of reasoning suggest that increased LFA may reflect shared disruptions of core brain functions across disorders rather than a disorder-specific mechanism. Consistent with this view, chronic pain, headache, fatigue, and depression frequently co-occur and share clinical features such as negative affect, cognitive dysfunction, and somatic symptoms [25]. Other neuropsychiatric conditions, such as schizophrenia or autism spectrum disorder, do not consistently show the same pattern [26, 27], suggesting that LFA increases may not represent a universal feature of neuropsychiatric disorders, but may instead relate to shared clinical features in certain conditions.

Several explanatory frameworks illustrate how such a transdiagnostic EEG finding can be mechanistically understood. At the systems level, the thalamo-cortical dysrhythmia (TCD) model attributes persistent low-frequency oscillations to altered thalamic pacemaking and disrupted thalamo-cortical coupling [28], a mechanism described across diverse neuropsychiatric conditions, including chronic pain [29]. At the circuit level, imbalances between excitation and inhibition, e.g., reduced GABAergic inhibition or excess glutamatergic drive, as reported in pain and mood disorders [30, 31], may bias cortical dynamics toward slower rhythms [32], thereby amplifying low-frequency activity. These alterations could involve both large-scale attentional networks and local cortical circuits, linking physiological changes to impaired sensory, cognitive, and emotional regulation. Since excitation and inhibition are central to gating and attentional control, sustained theta activity might reflect control mechanisms involved in regulating sensory and emotional processing [33]. From a clinical perspective, such mechanisms align with the concept of central sensitization, a state of heightened neural responsiveness that amplifies sensory signals and contributes to persistent symptoms [34]. Complementary computational accounts, such as predictive coding, propose that disruptions in the balance between top-down expectations and bottom-up sensory input lead to persistent misinterpretation of sensory signals [35], fostering symptoms such as chronic pain, fatigue, or other manifestations [36]. Since theta and beta oscillations play key roles in predictive processes, their abnormal increases may reflect maladaptive predictions across disorders [33, 37].

**Fig. 2:**
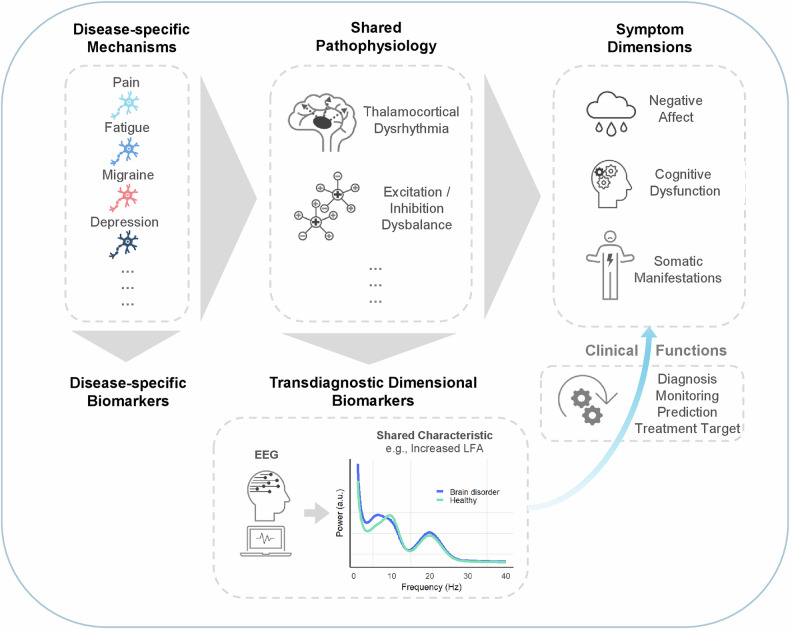
Conceptual framework illustrating increased low-frequency EEG activity as a transdiagnostic dimensional biomarker across brain-related disorders. The schematic depicts a cascade from disorder-specific mechanisms (left) to a shared neurophysiological signature (center) and symptom dimensions (right). Different conditions, such as chronic pain, migraine, chronic fatigue, depression, and other brain-related disorders, may converge on common pathophysiological processes. These might include thalamocortical dysrhythmia and excitatory–inhibitory imbalance. These hypothesized pathways can lead to EEG alterations, such as an increase in low-frequency activity (LFA; as shown in the simulated power spectrum at the bottom), reflecting a shared system-level characteristic rather than disorder-specific pathology. By directly measuring brain network dynamics, EEG lies close to the endpoint of the biological cascade that bridges molecular mechanisms with the clinical expression of shared symptom dimensions, making it uniquely suitable for transdiagnostic dimensional approaches. In this context, increased LFA serves as an example and potentially promising direction of research. Signals such as an increase in LFA hold potential clinical value as a transdiagnostic biomarker, supporting (i) monitoring of prognosis and treatment response, (ii) patient stratification for prediction, i.e., to identify individuals likely to benefit from specific interventions, (iii) susceptibility assessment for at-risk populations, and (iv) neuromodulation targeting (e.g., tES, rTMS) to normalize aberrant oscillatory dynamics.

Importantly, increased LFA is unlikely to reflect a single disorder-specific mechanism. Rather, it may index overlapping processes, including thalamo-cortical dysrhythmia, excitatory-inhibitory imbalance, and altered predictive coding, that converge on a vulnerable neural system. Accordingly, similar LFA patterns may emerge from converging underlying mechanisms across disorders. Resolving their relative contributions would require targeted experimental designs rather than cross-sectional inference. This convergence reinforces its potential as a transdiagnostic, dimensional biomarker: one that links mechanistic insights to shared symptom expressions without relying on categorical diagnoses (see Fig. 2). Notably, similar increases in LFA have been reported in neurodegenerative, neurodevelopmental, and psychiatric conditions [21–23], suggesting that LFA might index a more general dimension of brain health or vulnerability. Viewed through this lens, increased LFA exemplifies how mechanistic insights from EEG can inform the development of transdiagnostic, clinically actionable biomarkers that bridge neuroscience and clinical practice.

## From common EEG findings to clinically actionable biomarkers

Traditionally, biomarker research has focused on disorder-specific signals. However, the example of increased LFA in EEG illustrates that transdiagnostic, dimensional markers can be clinically useful precisely because they transcend diagnostic categories.

Such EEG biomarkers may serve multiple purposes, with their utility depending on systematic validation for the specific context. First, EEG biomarkers could enable disease monitoring, allowing clinicians to track prognosis, treatment responses, and disease course independently of categorical diagnoses. This would require that repeated measures accurately capture within-subject changes in symptoms. Second, EEG-derived phenotypes could support patient stratification, identifying subgroups more likely to benefit from specific interventions. Third, such markers may aid in risk identification and prevention. For instance, individuals with migraine who exhibit high theta activity may be at increased risk of developing depression, suggesting opportunities for early or preventive intervention. Likewise, people with chronic pain with pronounced low-frequency slowing may represent a subgroup prone to treatment resistance, warranting tailored strategies. Across these contexts, advancing toward clinical application will require benchmarking EEG biomarkers against established clinical measures.

Beyond their diagnostic or prognostic value, transdiagnostic EEG markers may also serve as therapeutic targets for neuromodulation. Interventions such as transcranial electrical stimulation (tES), repetitive transcranial magnetic stimulation (rTMS), or neurofeedback could be targeted at modulating abnormal low-frequency rhythms, paving the way for symptom-centered neuromodulation strategies across various disorders. Although the modulation of increased LFA has not been systematically explored in the disorders discussed here, evidence from other conditions suggests a potential precedent. In ADHD, an elevated theta/beta ratio appears to characterize a subgroup of patients [38]. In this context, targeting low-frequency alterations with neuromodulation approaches has been proposed as a possible strategy, although current evidence remains mixed [39, 40].

More broadly, the example of increased LFA illustrates a general principle: Given added predictive, monitoring, or stratification value beyond clinical markers, transdiagnostic, dimensional brain-based biomarkers can be clinically actionable without being disorder-specific. This framework extends beyond LFA to other EEG features (e.g., connectivity, aperiodic activity), other conditions and symptom dimensions (e.g., neurodegenerative disorders and cognitive deficits), and other biomarker modalities in and beyond functional brain imaging. Ultimately, moving beyond disorder silos toward mechanistically grounded, dimensional approaches may enable precision medicine in neuroscience, where biomarkers guide prevention, monitoring, and intervention across traditional diagnostic boundaries.

## Conclusions and outlook

Traditionally, the lack of disorder specificity of a biomarker has been seen as a limitation; here, we argue it is a strength. Transdiagnostic convergence provides a foundation for developing symptom-based biomarkers that are both mechanistically grounded and clinically actionable. Our example of increased LFA across chronic pain, headache, mood disorders, and related conditions exemplifies this potential, showing how EEG can capture shared neural alterations. These patterns of common neural dysfunctions may plausibly be related to symptom dimensions that cut across diagnoses.

The example of increased LFA illustrates how EEG biomarkers, when conceptualized on a transdiagnostic and dimensional level, can bridge basic neuroscience with clinical application. EEG is particularly suited for this task: it is cost-effective, scalable, and widely available across diverse clinical and research settings worldwide. Crucially, EEG provides a direct measure of brain function at the network level, placing it near the endpoint of the biological cascade that links genetic and molecular processes to symptom expression. Early steps in this cascade tend to capture more static disease-specific features, whereas network-level dynamics more closely reflect the real-time processes underlying cognition, emotion, and behavior. Consequently, measures of brain function, such as EEG, are particularly informative for transdiagnostic, dimensional approaches. Although limitations remain, such as low spatial resolution, intra-individual variability, and sensitivity to methodological choices and recording conditions, its accessibility and temporal precision make EEG a realistic and powerful candidate for translational biomarker development. In particular, power-based measures derived from eyes-closed recordings appear to be most reliable [23, 41].

Looking ahead, several priorities could accelerate progress. First, large-scale, transdiagnostic datasets that harmonize EEG and clinical data acquisition across neurological and psychiatric populations are essential. Such datasets could capture dimensional symptom patterns across conditions, enabling the identification of both shared and unique neural and clinical signatures. Second, longitudinal and interventional designs are needed to map EEG changes to symptom trajectories and treatment responses, enabling more personalized approaches. For example, these approaches could reveal EEG patterns that predict response to pharmacological or brain stimulation treatments. They could also uncover mechanisms underlying improvement or deterioration across neurological and psychiatric conditions. Third, multimodal integration with imaging or molecular markers could enhance both mechanistic interpretability and predictive power. For instance, linking EEG signatures to neurotransmitter function, white-matter integrity, or molecular pathology could clarify how cellular or biochemical changes shape large-scale brain dynamics and drive clinical symptoms. Furthermore, combining EEG with molecular or other imaging data can enhance the accuracy of models predicting clinical progression or therapeutic response. Together, these strategies can move EEG from a promising research tool to a robust, actionable biomarker platform.

The time is ripe to rethink EEG biomarkers, not as disorder-bound correlates, but as dimensional markers that connect neural mechanisms with clinical actionability, advancing the vision of precision medicine in neuroscience.
